# “Metastatic Cancer of Unknown Primary” or “Primary Metastatic Cancer”?

**DOI:** 10.3389/fonc.2019.01546

**Published:** 2020-01-17

**Authors:** Stefan Kolling, Ferdinando Ventre, Elena Geuna, Melissa Milan, Alberto Pisacane, Carla Boccaccio, Anna Sapino, Filippo Montemurro

**Affiliations:** ^1^Department of Investigative Clinical Oncology, Candiolo Cancer Institute, FPO-IRCCS, Candiolo, Italy; ^2^Multidisciplinary Oncology Outpatient Clinic, Candiolo Cancer Institute, FPO-IRCCS, Candiolo, Italy; ^3^Laboratory of Exploratory Research and Molecular Cancer Therapy, Candiolo Cancer Institute, FPO-IRCCS, Candiolo, Italy; ^4^Unit of Pathology, Candiolo Cancer Institute, FPO- IRCCS, Candiolo, Italy; ^5^Laboratory of Cancer Stem Cell Research, Candiolo Cancer Institute, FPO-IRCCS, Candiolo, Italy; ^6^Department of Oncology, University of Turin Medical School, Candiolo, Italy; ^7^Department of Medical Sciences, University of Turin, Turin, Italy

**Keywords:** cancer of unknown primary (CUP), primary metastatic cancer, metastasis, next generation sequencing, tissue of origin, targeted therapy, clinical trial, treatment

## Abstract

Cancer of unknown primary (CUP) is an umbrella term used to classify a heterogeneous group of metastatic cancers based on the absence of an identifiable primary tumor. Clinically, CUPs are characterized by a set of distinct features comprising early metastatic dissemination in an atypical pattern, an aggressive clinical course, poor response to empiric chemotherapy and, consequently, a short life expectancy. Two opposing strategies to change the dismal prognosis for the better are pursued. On the one hand, following the traditional *tissue-gnostic* approach, more and more sophisticated tissue-of-origin (TOO) classifier assays are employed to push identification of the putative primary to its limits with the clear intent of allowing tumor-site specific treatment. However, robust evidence supporting its routine clinical use is still lacking, notably with two recent randomized clinical trials failing to show a patient benefit of TOO-prediction based site-specific treatment over empiric chemotherapy in CUP. On the other hand, with regards to a *tissue-agnostic* strategy, precision medicine approaches targeting actionable genomic alterations have already transformed the treatment for many known tumor types. Yet, an unmet need remains for well-designed clinical trials to scrutinize its potential role in CUP beyond anecdotal case reports. In the absence of practice changing results, we believe that the emphasis on finding the presumed unknown primary tumor at all costs, implicit in the term CUP, has biased recent research in the field. Focusing on the distinct clinical features shared by all CUPs, we advocate adopting the term primary metastatic cancer (PMC) to denominate a distinct cancer entity instead. In our view, PMC should be considered the archetype of metastatic disease and as such, despite accounting for a mere 2–3% of malignancies, unraveling the mechanisms at play goes beyond improving the prognosis of patients with PMC and promises to greatly enhance our understanding of the metastatic process and carcinogenesis across all cancer types.

## Introduction

With more than 18 million new cases and 9.6 million deaths in the world in 2018, cancer is a formidable challenge for physicians, scientists, epidemiologists, healthcare providers, and most importantly, for patients and their families (1). In solid tumors, the death toll is almost invariably due to the development of systemic metastases that grow until they compromise the function of the colonized organs (2). Notwithstanding notable improvements achieved by targeted therapies and immunotherapy, curing or eradicating metastatic disease is still considered an unrealistic objective (3). Rather, inducing a chronic clinical course is pursued with the available treatments, and patients, apart from a few notable exceptions, remain on therapy as long as they can tolerate it, or until the tumor becomes treatment resistant. Historically, cytotoxic chemotherapy has been used to achieve this goal. More recently, biologically targeted therapies have changed the outlook of many metastatic disease settings, including big killers such as breast, lung, prostate colorectal cancer, and melanoma (3). Yet, metastatic disease still claims too many victims.

Excluding primary prevention, much of the current approach at reducing global cancer mortality still relies on two principles, both closely related to the concept of a “primary tumor.” The first is that prompt diagnosis of a tumor in one organ (primary tumor) before there is any clinical evidence (including diagnostic imaging) of colonization of other organs allows the only treatment that is potentially able to eradicate the disease, which is surgery. A proportion of patients who can have their primary tumor successfully resected will receive, in most instances, adjuvant local, and/or systemic treatments with the aim of neutralizing undetectable micrometastatic disease. The second principle is that the site of the primary tumor, or its organ derivation, is a key determinant in guiding therapeutic choices. This is a heritage of years of valuable research with chemotherapeutic agents, where different classes of compounds were associated with variable efficacy in cancers of different origins, whether they be used in the adjuvant setting or to treat metastatic disease. Strategies to exploit these two principles have paid off, as the constant decline in cancer mortality observed in Western countries demonstrates (4, 5). As a matter of fact, currently available international guidelines are mostly “tissue gnostic,” presenting anticancer treatments by primary tumor site of origin. Precision medicine is revealing that some druggable molecular targets are present in different types of tumors, leading to the approval of the first “tissue-agnostic strategies” (6). Despite a growing number of such successfully druggable molecular alterations, cancer taxonomy is still heavily organ oriented rather than based on molecular profiling.

Having discussed these two principles, it is easy to understand how dramatic a diagnosis of “Cancer of Unknown Primary” (CUP) must be for a patient, a metastatic cancer presentation that accounts for about 2–3% of the several million new cancer cases diagnosed every year in the world (7–9). CUPs are usually diagnosed in patients with severe and rapidly worsening metastasis-related symptoms. Furthermore, the organ of origin cannot be reliably determined combining clinical (including imaging and endoscopy) and histopathological (including classical immunohistochemical biomarkers) evaluations. As a consequence, CUPs that have an epithelial derivation (most of them), are treated by empiric chemotherapy and the overall survival of about 12 months has remained virtually unchanged over the last decades (9–11).

Here we review the current knowledge on the epidemiology, diagnosis and treatment of CUP as well as summarize recent advances in CUP research. Furthermore, we propose a different definition of CUP as a distinct clinical entity that is the archetype of metastatic cancer, with the focus on unraveling the key mechanisms underlying the metastatic process across all tumor types.

## Definition and Epidemiology of Metastatic Cancer of Unknown Primary

**Figure 1:**
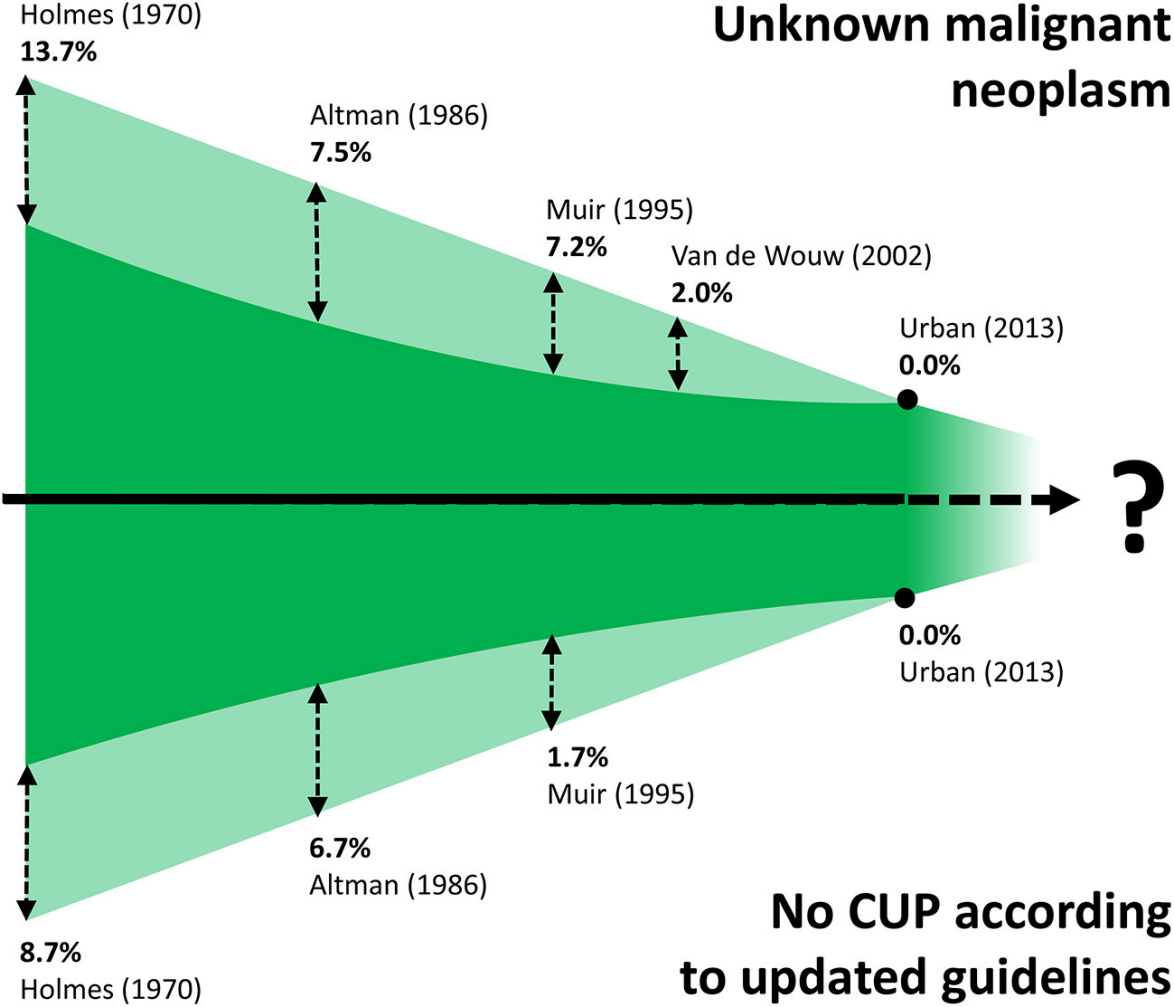
Schematic (not to scale) illustration of the difficulty of comparing CUP studies from different decades. Improvements in immunopathologic diagnostics has led to the gradual disappearance of CUP cases previously classified as *unknown malignant neoplasms* (shaded area at top, with proportions of cases that would be excluded with current diagnostics). Refinement of CUP guidelines (10, 11) means that a proportion of historic CUP cases would be excluded nowadays, such as tumors of mesenchymal origin, and melanomas (shaded area at bottom, with proportions of cases that would be excluded by applying current guidelines).

Cancer of unknown primary is a clinical presentation of cancer with systemic metastases that cannot be attributed to a precise organ of origin beyond reasonable doubt. Current guidelines provide criteria to define and stratify CUPs, using “*ad excludendum*” algorithms (9–11). Fundamentally, the definition of CUP contains two elements. Firstly, checking organs by clinical examination, diagnostic imaging and endoscopy does not reveal a primary tumor in one organ of origin. Secondly, beyond clarifying the epithelial or mesenchymal origin, conventional immunohistochemistry analysis does not identify characteristics that can clearly relate to a tissue of origin (TOO). Intuitively, the ability to identify a TOO by combining the findings of clinical and histopathological evaluation has changed over time due to constant improvements in the repertoire of imaging and histopathological techniques. Hence, by the very nature of the CUP definition, it is intrinsically difficult to provide accurate incidence data and, even more so, to compare studies from different decades (Figure 1) (8, 12–15).

**Figure 2:**
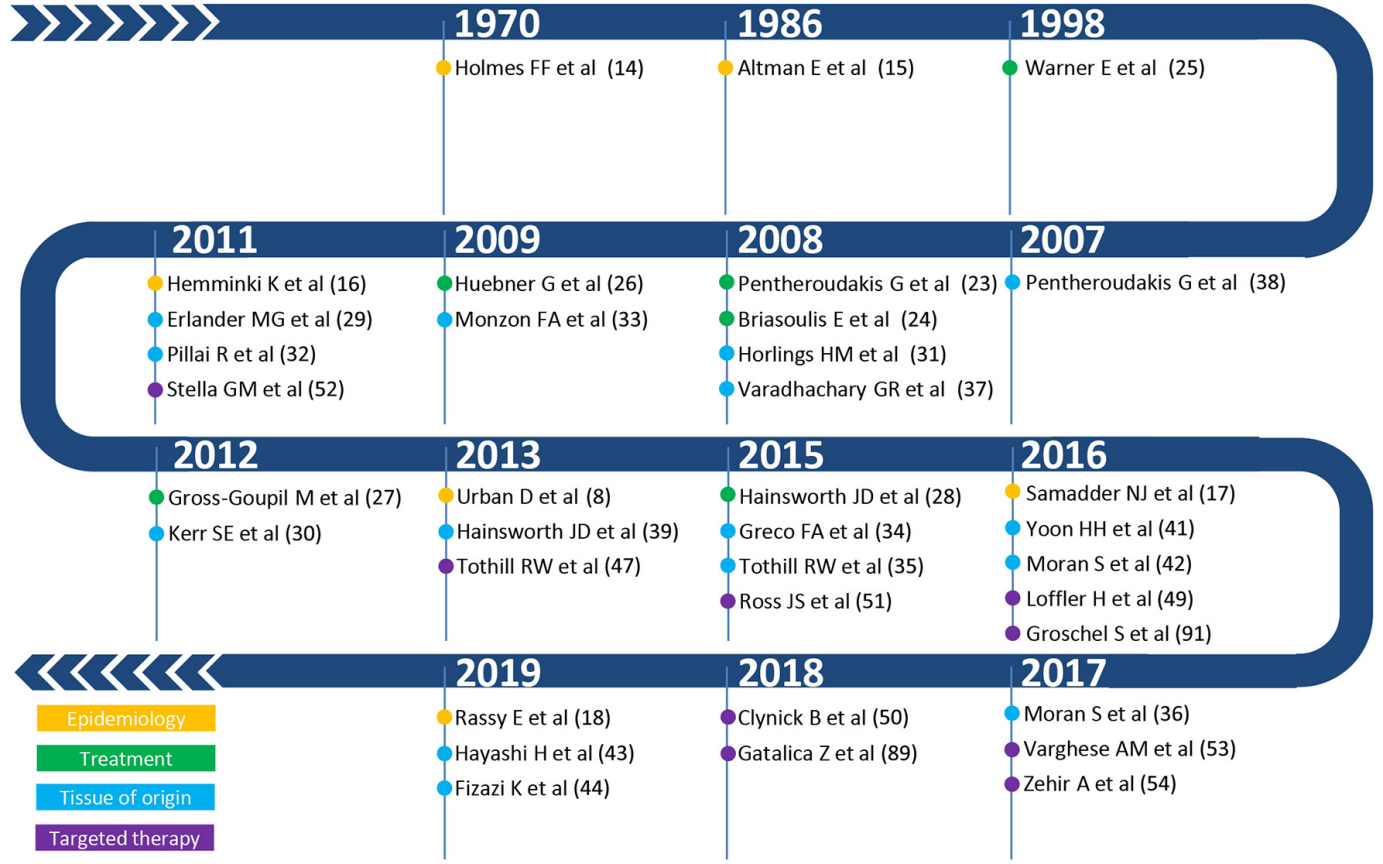
Summary of the most relevant publications on epidemiology (yellow), therapeutic management (green), tissue of origin (light blue), and targeted therapy (purple) in CUP.

One of the first occurrences of the term Metastatic Cancer of Unknown Primary was in a paper published by Holmes and Fouts in the journal Cancer in 1970 (Figure 2) (14). The authors analyzed the tumor registry of the Kansas Medical Center between 1944 and 1969. Out of a total of 21,000 consecutive patients registered in that time frame, 686 patients (3%) were identified with metastases from unknown primary cancers. The yearly incidence changed by little (ranging from 2.1 to 4.6%), trending toward an increase in the last years of collection, suggesting no precise role of the improvement in diagnostic techniques that may have occurred in the selected time interval. Adenocarcinoma, carcinoma, anaplastic carcinoma, and squamous cell carcinoma accounted for 79% of all histopathological diagnoses, whereas about 4% of the tumors were of mesenchymal origin. It is worth noting that according to current guidelines tumors of mesenchymal origin, among others, are not anymore considered nor treated as CUPs, a factor further complicating the comparison of historic CUP studies as illustrated in Figure 1. Seventy-fifth percentage of the patients died from metastatic progression within 1 year from diagnosis and an additional 11% died during the second year. Long-term survivors were identified in only 5%. Similar results were presented by Altman et al. who analyzed the tumor registry of the Yale New Haven Hospital from 1922 to 1981 (15). The incidence of CUP was approximately constant over the decades covered by the registry with about 3%. Overall survival was poor, with a median duration of 5 months. The proportion of patients dying within one and 2 years from diagnosis was 75 and 88%, respectively. Histology findings were consistent with those previously reported by Holmes. More recently, Urban et al. analyzed the Surveillance, Epidemiology, and End Results registry between 1973 and 2008 with the aim of reporting temporal trends and outcome in 106,641 CUP patients (8). The proportion of cancer diagnosed as CUP amounted to about 4% in the early 70′ and unlike in the former two studies, steadily decreased over time down to an estimated 2% in 2010. Once again, most patients died within 1 or 2 years from diagnosis. Squamous cell and neuroendocrine histology were associated with longer median survival, however not exceeding 15 months. Interestingly, squamous cell histology was associated with a temporal trend toward improved prognosis, whereas median overall survival remained 3 months for non-squamous CUPs across all groups stratified according to decade of diagnosis. In a first-of-its-kind study, Hemminki et al. investigated the role of familial risk in CUP, analyzing the data of the Swedish Family-Cancer Database from 1958 to 2008 (16). In total, around 3% of all CUP cases showed a defined pattern of familial clustering. Notably, an increased risk of CUP in the offspring generation was associated with the presence of—in decreasing order—lung, kidney, liver, ovarian, colorectal, and breast cancers and melanoma in the parent generation. Based on their results, the authors suggest that CUP may be a disease of a defined set of cancers which share a marked genetic background and metastatic mechanisms rather than a disease of random metastatic cancers. In this view, the associated familial sites listed above are likely sites of the occult origin for CUP. Another epidemiological case control study on familial cancer clustering conducted using the Utah Cancer Registry (UCR) supports the hypothesis of a common genetic background in CUP (17). Samadder et al. determined an increased risk of CUP and of a series of other epithelial cancers in first-degree, second-degree relatives and first cousins of index cases with a confirmed diagnosis of CUP. The association in terms of relative risk varied across different cancers and degree of parenthood and was, in general, not very strong, ranging from as low as 1.06 for colon cancer in first cousins and 1.43 for lung cancer in first-degree relatives. For these and other inherent reasons, including the rarity of this entity, the molecular pathogenesis of CUP remains largely to be elucidated and the implications of “familial CUP” is still an investigational subject (18).

**Figure 3:**
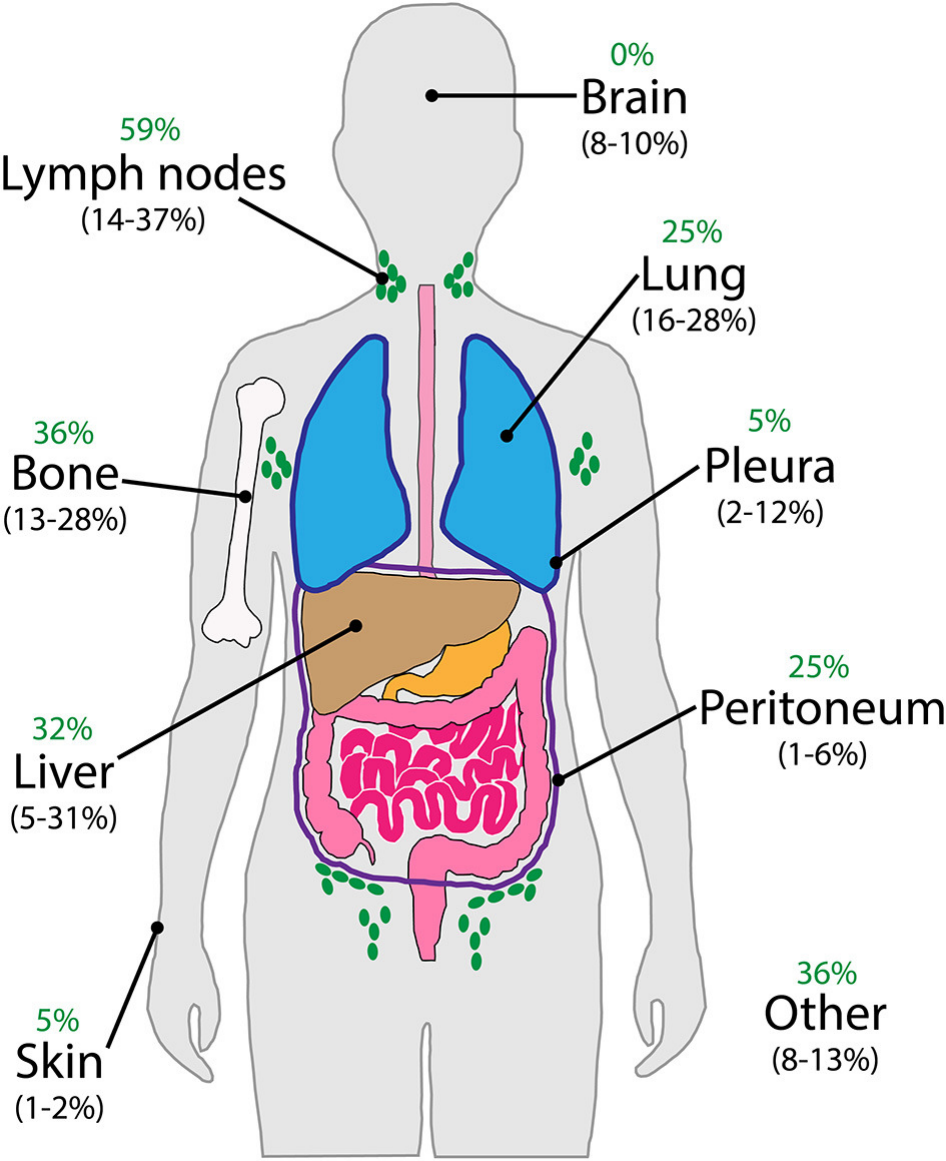
Major sites of metastatic involvement on initial presentation of CUP patients. Comparison of data from three published historic cohorts (in brackets) (20–22) with that of 44 patients currently enrolled in our ongoing clinical trial (green).

With regards to the clinical presentation of patients with CUP, by definition, the signs and symptoms are manifestation of the neoplastic involvement of the metastases. In the literature, the incidence of the most frequently encountered presenting metastatic sites varies with patient selection criteria and thoroughness of the diagnostic work-up over the decades (19). Figure 3 illustrates the major sites of initial metastatic presentation in three historic CUP cohorts (20–22) in direct comparison with those of 44 CUP patients recently enrolled in an ongoing clinical trial at our institution. Despite apparent numerical differences for each individual site, involvement of lymph nodes, bone, liver and lung is most common overall, with other sites such as brain, pleura, peritoneum, and skin being less frequently observed. It is worth noting that, unlike for the historic cohorts, none of our patients had cerebral metastases on initial presentation, but some developed them later on during disease progression. Intuitively, this may be explained by the fact that the disease is likely diagnosed at an earlier stage nowadays.

## Current Therapeutic Management of CUP

Once the diagnosis of CUP has been confirmed, the objectives of management are those of metastatic cancer in general, resorting to best supportive care only in those patients with prohibitive performance status (9–11). A favorable subset of CUPs accounts for about 15% of all new diagnoses and carries a better prognosis because histology and pattern of disease are largely reminiscent of a cancer of known origin or of a potentially equal tumor (9–11). Examples are regionally limited neck metastases from squamous cell carcinoma, or peritoneal carcinomatosis from serous papillary histology types in female subjects. When treatment is feasible, median overall survival in this favorable group ~15–20 months. Unfortunately, most newly diagnosed cases belong to what is termed the unfavorable subset. According to a prognostic stratification based on performance status and pre-treatment lactate dehydrogenase (LDH) levels, median overall survival of these patients with unfavorable prognosis ranges from 4 to 10 months (9). These figures have remained substantially unchanged over the years despite the availability of new chemotherapeutic agents. This is also reflected in the latest NCCN recommendations that list 11 polychemotherapy regimens for adenocarcinoma histology, most of which include a platinum agent, and nine platinum-containing regimens for squamous histology (11). The vast majority of these regimens, which frequently contain taxanes, gemcitabine and irinotecan, are based on results from single-arm, phase II data to support their use in CUP at best (23–25). Additionally, data from randomized studies are scarce and mostly come from small trials providing suggestions rather than clear guidance regarding preferred regimens (26–28). For example, the superiority of polychemotherapy compared to single agent chemotherapy is an open issue in CUP. The only trial testing this hypothesis was closed prematurely because of slow accrual (27). In the enrolled population of about 50 patients, no clear PFS advantage emerged from comparing cisplatin and gemcitabine vs. cisplatin alone. Regarding biologically targeted therapies, a single randomized trial evaluated the addition of the histone deacetylase inhibitor belinostat to paclitaxel and carboplatin (28). In this small, phase II randomized trial, belinostat did not prolong PFS, which was the primary study endpoint, but was associated with increased response rate. In summary, while empiric chemotherapy has been the mainstay of treatment of CUP patients with a permissive performance status for years, the clinical outcome remains largely unsatisfactory.

## Could the Management of CUP Be Improved?

Newer classes of chemotherapeutic agents introduced over the years have led to measurable improvements in patient outcomes in some metastatic cancers of known origin. For example, this is the case for breast, ovarian, lung, or colorectal cancer and malignant melanoma (3). Having better and multiple agents at hand to be used sequentially could result in longer metastatic disease control. For this reason, a more accurate identification of the organ of origin of CUP has been pursued as a strategy to improve management for years. Recently, this field has witnessed a notable expansion due to the availability of the “-omics,” including genomic, transcriptomic, epigenomic, and proteomic analysis. Based on these technological innovations, two novel emerging routes will be addressed in this chapter: (1) chasing the identification of the tissue of origin or (2) finding actionable mutations regardless of the putative tissue of origin.

### Chasing the Tissue of Origin (TOO) in CUP

CUP is a diagnosis of exclusion that holds true as long as the primary tumor remains elusive. As the failure to detect a primary cancer in one organ of origin is strongly correlated with the technical limitations of the diagnostic procedures at hand, the term CUP might be no more than a convention: an umbrella term used to artificially classify a very heterogeneous group of biologically different types of cancers, the biology of each dictated by the hidden primary. In this TOO-oriented view, it appears imperative to push the molecular characterization to its limits in order to identify a putative TOO at all costs and to treat CUPs as a high-grade metastatic tumor of the respective TOO. Based on this assumption, molecular methods using gene expression profiling, gene microarrays, microRNA, and DNA methylation analysis are increasingly employed as classifier assays to predict the TOO. Conceptually, the TOO classifiers are trained on a database of TOO-specific molecular signatures established by analyzing primaries of known origin and can predict the TOO of known primaries with accuracies of 76–96% (29–35). As already pointed out elsewhere, an important limitation of this approach is the number of different tumor types used in training the TOO classifier (36). In retrospective studies, molecular classification yielded a TOO prediction that was consistent with clinical and pathologic features in the majority of CUP patients (31, 37). Applying TOO classifiers to CUP specimens, a putative primary origin could be predicted in 83–90% of cases. However, due to the very nature of the disease, no primary tumor exists to validate the reliability of such predictions for CUP during the patient's lifetime. Retrospective attempts to verify predictions in CUP using the findings of post-mortem autopsy series have revealed discrepancies in the spectrum and relative proportions of primaries compared with molecularly assigned putative TOOs (38, 39), reviewed in more detail by Bochtler and Krämer (40). Notably, lung and pancreas primaries were more likely findings on autopsy, whereas liver, biliary tract, urothelial, and breast primaries prevailed according to TOO classifiers. A prospective study to systematically verify TOO predictions in autopsied patients would therefore be highly desirable. Since TOO classifiers are based on similarities rather than differences between CUPs and the training set of known primary tumors, a CUP with a molecularly assigned TOO may still be biologically distinct from its counterpart of known primary and hence the generally assumed benefit of site-specific treatment must be proven in clinical trials. As reviewed by Moran et al., a handful of clinical trials have evaluated the impact of TOO-prediction based site-specific treatment with regards to improved clinical outcome (36). In a small prospective phase II trial, Yoon et al. successfully molecularly profiled the TOO of 38 out of 45 confirmed CUP patients with a 2000-gene expression microarray-based assay (41). All of these patients were treated with carboplatin, paclitaxel and everolimus. Stratification of patients according to predicted TOO showed significantly longer progression-free survival (PFS) and overall survival (OS) in patients with tumor types known to be platinum/taxane responsive compared with those with platinum/taxane resistant tumors (PFS 6.4 vs. 3.5 months; OS 17.8 vs. 8.3 months). In a larger, but non-randomized, study by Hainsworth et al., 194 patients received site-specific treatment directed by the respective TOO prediction using a 92-gene RT-PCR classifier (39). In these, the median OS reached 12.5 months, which compared favorably to historic trial data (OS 9–10 months). Interestingly, survival times differed significantly between those patients predicted to have more responsive tumor types and those predicted to have less responsive tumor types, respectively (OS 13.4 months vs. 7.6 months). In addition to gene expression profiling, Moran et al. advocate predicting the TOO by analyzing DNA methylation profiles of CUP (42). Their epigenetic classifier was trained on a set of 2,790 samples of 38 known distinct tumor types and subsequently validated using an independent set of 7,691 known samples of the same tumor types (97.7% sensitivity, 99.6% specificity). Applied to samples of confirmed CUP cases, a putative primary site could be predicted in 87% of the patients. Importantly, the soundness of these predictions was successfully verified by either autopsy examination, more comprehensive immunohistochemical analysis guided by the TOO prediction or identification of the primary at a later stage during follow-up. Albeit retrospectively, they found that overall survival appeared to be significantly longer for those patients who had received site-specific treatment in line with their epigenetic TOO prediction compared with those patients who received empirical therapy that did not match the TOO prediction (OS 13.6 months vs. 6 months). These results have led to the first calls for a paradigm shift to support the routine use of molecular tumor profiling in the standard management of patients with CUP. In stark contrast to this, less promising evidence has recently come from two prospective, randomized trials. Hayashi et al. randomly assigned 101 previously untreated patients of the unfavorable CUP subset to either receive standard site-specific therapy as predicted by microarray analysis or empirical paclitaxel and carboplatin chemotherapy in a phase II trial (43). Site-specific treatment did not result in a significant improvement in 1-year survival rate, OS or PFS. Patients predicted to have more responsive tumor types showed longer survival compared with those predicted to have less responsive tumor types (OS 16.7 months vs. 10.6 months). Even though this differential survival of patients with more responsive tumor types did not translate into an improved OS for those treated with site-specific therapy vs. empirical therapy, it highlights the potential prognostic value of TOO prediction, a finding consistent with a previous trial (39). Similarly, Fizazi et al. conducted an even larger prospective phase III trial with 223 treatment-naïve CUP patients with unfavorable prognosis randomized to either site-specific therapy directed by comprehensive molecular gene expression analysis or empiric chemotherapy (44). Once again, site-specific therapy did not improve clinical outcome as assessed by 1-year survival, PFS and OS. Due to this lack of robust evidence from randomized, prospective data clearly demonstrating improved clinical outcomes for TOO-prediction based site-specific treatment, international guidelines do not currently advocate for routine prediction of the TOO in CUP patients (11).

### Moving Beyond the TOO in CUP Toward Targeted Therapy

On the other end of the spectrum, there is the opposing approach of moving beyond the traditional cancer classification chiefly based on organ type and TOO. Instead, an underlying CUP-specific pro-metastatic molecular signature is postulated to explain the distinct clinical features shared by most CUPs, such as an aggressive clinical course, early metastatic dissemination in an atypical pattern, and poor response to treatment. In this mindset, treating CUP based on a putative primary site will not improve clinical outcomes. Rather, unraveling said still elusive CUP-specific signature is expected to delineate driver genetic alterations as new targets for a precision medicine approach (45, 46).

#### Targeted Therapy

A handful of descriptive studies have examined next-generation sequencing (NGS) in confirmed CUP patients, with 52–85% of patients found to harbor potentially actionable genomic alterations (47–51). In a study of 200 CUP specimens, Ross et al. revealed a median number of 4.2 genomic alterations per patient (51). The most commonly affected genes potentially affecting treatment decisions included KRAS (20%), CDKN2A (19%), MCL1 (10%), PTEN (7%), PIK3CA (9%), and ERBB2 (8%), whereas the most common biologically relevant alterations that cannot currently be linked to targeted treatment were found in TP53 (55%), MYC (12%), ARIDIA (11%), SMAD4 (6%), SMARCA4 (6%), and RB1 (6%). Remarkably, actionable mutations in the receptor tyrosine kinase/Ras signaling pathway were nearly twice as common in adenocarcinoma CUP (72%) compared with non-adenocarcinoma CUP (39%). Upstream of the Ras signaling pathway, Stella et al. found an unexpectedly high incidence of somatic MET mutations in CUP compared with tumors of known primary site (30 vs. 4%), possibly linking the CUP phenotype to inappropriate activation of the invasive growth program (52). More recently, Varghese et al. made the case for a more refined definition of “potentially actionable” alterations on the basis of varying levels of evidence supporting targeted therapies as reported in the OncoKB database, a curated knowledge base of the functions and treatment implications of somatic mutations (53). According to their stricter selection, only 30% of CUP patients had potentially targetable genomic alterations identified by molecular profiling, a finding they verified to be consistent with the aforementioned studies when applying the same criteria. Making use of the same criteria, Zehir et al. prospectively sequenced samples of 186 CUP patients as part of a large-scale characterization of the mutational landscape of advanced metastatic cancer across tumor types and confirmed a frequency of potentially targetable genomic alterations in the order of 30% (54). Interestingly, their MSK-IMPACT test stands out in that it also captures the promoter of TERT, a region not typically covered by whole-exome-sequencing analysis. Mutations in the TERT promoter have been shown to lead to upregulated telomerase expression and evasion of apoptosis (55, 56). The authors confirmed a high prevalence of these mutations in patients with advanced solid tumors, being present in 9% of analyzed CUP samples among others, and noted a general trend toward shorter survival, consistent with the literature (57–59). With the advent of techniques in clinical practice able to extract circulating tumor DNA (ctDNA) from liquid biopsies, molecular profiling has not only become safer, easier, and cheaper than with invasive tissue biopsies, but also circumvents the problem of tumor heterogeneity by capturing genes from multiple metastatic sites (48, 60). Moreover, these characteristics of liquid biopsies render ctDNA the ideal tool to monitor for dynamic changes in the mutational profile of the tumor as patients receive cancer therapy and a potential role of liquid biopsies in the clinical workflow for CUP has been proposed (61). While more and more data on potentially targetable alterations in CUP keeps emerging, it is crucial to stress that the efficacy of targeted agents varies widely across solid tumor types and their value in CUP is uncertain without a TOO. The most dramatic example of this is the variable response to BRAF inhibitors across different tumor types, ranging from a high response in BRAF V600E-mutated melanoma to a complete lack of activity of such agents in BRAF V600E-mutated colorectal cancer (62–64). Hence, a completely TOO-agnostic treatment approach is unlikely for most targeted agents. To date, documented responses to targeted therapy in CUP patients are limited to anecdotal descriptions of case reports at best (Table 1) (65–78). Similarly, available evidence from several basket trials for advanced metastatic cancer, including a subset of CUP patients, is weak. The multi-center SHIVA trial was the first prospective, randomized phase-II study on any kind of metastatic solid tumor to address the question whether targeted therapy does improve PFS compared with standard chemotherapy (79). This trial was limited to targeting alterations in three distinct molecular pathways (hormone receptors, PI3K/AKT/mTOR or RAF/MEK) in 195 of 741 patients (26%) screened. Median PFS was not significantly prolonged compared with the control group (2.3 vs. 2.0 months; hazard ratio 0.88, 95% CI 0.65–1.19, *p* = 0.41). However, the patient cohort included only 5 CUPs and firm conclusions should not be drawn. In a similar fashion, Massard et al. screened a total of 1,035 patients with advanced metastatic cancers for potentially targetable alterations in the prospective single-arm open-label MOSCATO 01 trial and assessed the clinical benefit of the respective targeted treatment (80). The primary study endpoint was a growth modulation index (GMI) > 1.3. The GMI is the ratio between the time to progression with the investigational treatment and the time to progression with the last treatment received before study entry (81). While a GMI > 1.3 was indeed observed in 33% of patients receiving targeted therapy, implying an improved outcome in a subset of patients, this subset accounted for only 7% of 948 successfully screened patients overall. In particular, out of 36 screened CUP patients, none of the 5 CUP patients eventually treated benefited from this approach in terms of GMI.

**Table 1:** Case reports of patients with cancer of unknown primary responding to targeted therapy.

**References**	**Age (y)/Sex**	**Diagnosis**	**Metastatic site**	**Genetic alterations**	**Treatment**	**Outcome**
Asakura et al. (65)	55/female	Poorly differentiated adenocarcinoma	Axillary and neck lymph nodes	HER2 overexpression	Trastuzumab, vinorelbine	CR
Yamada et al. (66)	53/male	Adenocarcinoma of unknown primary	Cervical lymph nodes, bone, bilateral adrenal glands	EGFR mutation	Gefitinib	PR
Tan et al. (67)	50/male	Poorly differentiated adenocarcinoma	Neck, retropectoral, and axillary lymph nodes	EGFR mutation	Gefitinib	PFS (11 months)
Chung et al. (68)	53/female	Poorly differentiated adenocarcinoma	Subcutaneous right upper extremity nodules, lung, subcranial, and mediastinal lymph nodes	ALK-ELM4 rearrangement MCL1 amplification CDKN2A/2B deletion	Crizotinib	CR
Palma et al. (69)	59/female	Poorly differentiated adenocarcinoma	Cerebral metastasis, left mid-abdominal mass	MET amplification CCND1 amplification MYC amplification KRAS mutation TP53 mutation CARD11 mutation	Crizotinib	CR PFS >19 months
Whang et al. (70)	60 s/man	Poorly differentiated adenocarcinoma	Bone, lymph nodes	ErbB2 amplification BRCA1 mutation	Trastuzumab, lapatinib, chemotherapy	PR PFS > 24 months
Hainsworth et al. (71)	45/female	Signet-ring cell adenocarcinoma	Mediastinum, adrenal glands	ALK-ELM4 rearrangement	Crizotinib	PR PFS > 40 months
Shima et al. (72)	68/female	Adenocarcinoma of unknown primary	Supraclavicular and axillary lymph nodes	HER2 overexpression	Trastuzumab, chemotherapy	CR
Kato et al. (48)	82/male	Adenocarcinoma of unknown primary	Liver and abdominal lymph node metastases	KRAS mutation MLH1 mutation	Trametinib Nivolumab	PR
Røe et al. (73)	62/woman	Undifferentiated carcinoma	Inguinal lymph nodes	BRAF mutation	Ipilimumab	CR PFS (52 months)
Yamasaki et al. (74)	67/male	Adenocarcinoma of unknown primary	Brain, supraclavicular, mediastinal, and upper abdominal lymph nodes	EGFR mutation ALK rearrangement c-ROS1 rearrangement	Erlotinib	PR PFS (8 months)
Taniwaki et al. (75)	55/female	Poorly differentiated adenocarcinoma	Laterocervical, abdominal, bilateral supraclavicular, and mediastinal lymph nodes	ROS1 rearrangement	Crizotinib	PR PFS (3 months)
Yao et al. (76)	67/male	Poorly differentiated adenocarcinoma	Laterocervical and mediastinal lymph nodes	NTRK1 mutation CCDN1 amplification TP53 mutation MET amplification	Crizotinib	PFS (8.5 months) OS (10 months)
Zhao et al. (77)	31/female	Poorly differentiated adenocarcinoma	Liver, lung, and mediastinal lymph nodes	ALK-ELM4 rearrangement	Crizotinib, brigatinib	PR

*PFS, progression free survival; CR, complete response; PR, partial response; OS, overall survival*.

These results were essentially confirmed by the more recent ProfiLER trial, in which 27% of 2,579 patients had recommendations for targeted treatment, but only 6% of patients actually received such treatment, with an overall response rate of a mere 0.9% (82). In the cohort, 11 of 43 CUP patients had potentially targetable alterations, five of which were treated accordingly but showed no response.

Further insight into the role of comprehensive molecular profiling in guiding treatment decisions of CUP patients could emerge from large-scale prospective clinical trials either in metastatic cancer patients across tumor types, or preferably, from trials specifically focusing on CUP patients. In terms of currently ongoing basket trials, the MATCH (NCT02465060) and TAPUR studies (NCT02693535) investigate efficacy and safety of the targeted approach in patients with advanced cancer. The CUPISCO trial (NCT03498521), a phase II randomized study, is of particular interest as it evaluates the efficacy and safety of targeted therapy vs. standard chemotherapy exclusively in CUP. The results of these trials are eagerly awaited to shed more light on the role of targeted therapy in CUP.

#### Immunotherapy

In the wake of the success of checkpoint inhibitors in the treatment of several cancer types in the metastatic setting, these may be reasonably expected to have an impact on clinical outcome in patients with CUP as well. Reliable markers need yet to be established to predict responsiveness to immunotherapy and to identify the subset of patients expected to benefit the most. The most promising markers currently under study comprise microsatellite instability (MSI), high tumor mutational load (TML) (83, 84), mismatch repair deficiency (dMMR) (85), HLA class-I diversity (86, 87), and biomarker expression (e.g., PD1, PD-L1) (88). Screening 389 cases of CUP, Gatalica et al. found that 28% of samples were positive for one or more of said predictive markers (89). In this context, it is worth mentioning that the anti-PD1 antibody pembrolizumab has recently become the first drug to have gained FDA approval for entirely TOO-agnostic treatment of any tumor with confirmed high MSI and/or dMMR (90). To the best of our knowledge, there are currently only two isolated case reports of response to immunotherapy in one CUP patient with high PD-L1 expression (91), and another one with confirmed dMMR (48) published in the literature to date. However, a range of four single-arm phase II clinical trials are ongoing to scrutinize the role of immunotherapy in the context of CUP, with two of them recruiting treatment-naïve patients only (NCT03391973, NCT03752333) and the other two recruiting pre-treated patients after progression (NCT02721732, NCT03396471). On the current basis, we feel that the results from these studies must be awaited to validate the listed predictive markers and to exploit the full potential of immunotherapy in CUP.

## Metastatic Cancer of Unknown Primary (CUP) or Primary Metastatic Cancer (PMC)?

Over the years, a diagnosis of CUP has mainly reflected the inability of finding a primary organ of origin. CUPs break the canonical paradigm of linear progression, i.e., that a tumor originates and grows in a tissue, acquires the competence to disseminate and seeds to distant tissue, and after a latency of months or years, surfaces as overt metastatic disease. Our current therapeutic approach at treating cancer is based on this paradigmatic model, where early diagnosis, surgery, and adjuvant treatments aimed at eradicating micrometastatic disease have proven to successfully improve patient outlook. Newer imaging and molecular diagnostic tools have shown to impact on the ability to identify the TOO in CUP, with a consequent drop in the incidence of CUP. Although 5–10% of CUP patients are still missing their primary organ of origin, it is conceivable that soon this gap may be filled by newer technologies. Hence, it is possible that the term CUP will no longer have any reason to exist. Granted this prediction, the real question is: “does this really matter for patients”? Thus, far, the few available clinical trials that exploited improved prediction of the TOO have not provided practice changing results. In addition, precision medicine can be applied to patients with CUP in a similar way to metastatic cancers of known origin, but no CUP-specific molecular target has been found yet. Furthermore, and in more general terms, matching molecular alterations with an effective drug is still far from yielding major improvements in metastatic cancer patients and even less so in CUP. Timing of test results, target prioritization, availability of the portfolio of drugs to cover the possible spectrum of molecular alterations in a timely fashion and the fact that not all the molecular targets are druggable in a tissue agnostic way are just a few of the hurdles that this approach is facing (92).

With this in mind, we believe that the emphasis on finding the primary tumor that is implicit in the term CUP has biased recent research in the field of this enigmatic disease. In fact, this research has been conducted with the valid aim of improving patient outlook, but unfortunately, so far, it has largely failed in this intent. Therefore, we propose to abandon the term CUP and to focus on the set of distinct clinical features shared by the unfavorable CUP subset: aggressive clinical course, early metastatic spread by poorly differentiated cancer cells, resistance to available anticancer drugs, and short life expectancy from the time of diagnosis. The genetic background allowing cancer cells to migrate and colonize distant organs is likely acquired very early during cancerogenesis and must be defined. Possibly, this occurs in a cancer stem cell that has an intrinsic ability to migrate, seed, and home to different organs (93). An immunologically permissive host microenvironment may also play a role in the widespread dissemination that is characteristic of poor-prognosis patients. For this reason, we believe that the term “Primary Metastatic Cancer” (PMC), coined by Pentheroudakis et al. (94), more accurately describes the essence of this genetically determined syndrome. Here, the term “primary” does no longer refer to a putative lineage or organ of origin, which would call for a tissue specific management approach. Rather, it refers to the ability to spread systemically well-ahead of local outgrowth, an ability that, as stated, must have a genetic background regarding the tumor, the host, or, most likely, both. With PMC being the archetype of metastatic disease, unraveling the genetic and epigenetic mechanisms underlying the hypermetastatic phenotype may have paramount implications for cancer prevention, diagnosis and treatment. To date, advances in understanding the biology of CUP and identifying such mechanisms have been weak, as reviewed in detail elsewhere (94–96). Frequent chromosomal aneuploidy and abnormalities [e.g., in the short arm of chromosome 1; isochromosome i(12p)] as well as a high incidence of overexpression and mutations in common tumor suppressor genes (e.g., p53) and oncogenes (e.g., c-Myc, Ras, Her-2, c-Kit, PIK3CA) have been reported. However, considerable heterogeneity between patient cohorts from different groups is present and most of these findings occur at the same rates reported in metastatic tumors of known primaries. Similarly, the prognostic significance of angiogenesis in CUP is unclear. Reports of both increased microvessel density (97) and promising clinical results of antiangiogenic treatment (98, 99) on the one hand are in conflict with the observation of a low angiogenic phenotype in CUP on the other hand (100). Progress in this field is hampered by both the low overall frequency of CUP cases and the general scarcity of biological material obtained from single patients. Hence, we believe that there is a strong and yet unmet need for the generation of dedicated patient-derived *in-vivo* and *in-vitro* models to advance our knowledge of the biology of CUP. Pursuing this approach, we launched the Agnostos program, a clinical and translational platform seeking to determine the molecular basis of the hypermetastatic phenotype and identify potential targets for targeted therapies. The Agnostos program includes a prospective, randomized phase-II clinical trial (NCT02607202) and the Agnostos Profiling study, both ongoing and systematically collecting clinical annotations, tumor biopsies and other biological materials from enrolled patients. The clinical trial assesses the efficacy of nab-paclitaxel in combination with either carboplatin or gemcitabine, respectively, in rigorously selected patients of the unfavorable CUP subset with respect to the objective response rate as primary study endpoint. The trial is complemented by the Agnostos Profiling study, namely including our efforts concentrated on establishing patient-derived xenopatients as well as *in-vitro* models from tumor biopsy samples and circulating tumor cells for the identification of molecular targets and of potential therapeutic strategies in the preclinical and clinical setting. From this program we expect to derive substantial information on the culprits of the metastatic process and to hypothesize strategies aimed at designing interventional trials.

## Author Contributions

FM: conception and design. SK, FV, MM, and FM: writing of manuscript and analysis and interpretation of data. EG, CB, AP, AS, and FM: review and revision of manuscript. SK and FM: final approval of the version to be submitted.

### Conflict of Interest

FM has received Speaker's or Advisory Board Honoraria from Roche, Novartis, Pfizer and Lilly and travel grants from Roche. The remaining authors declare that the research was conducted in the absence of any commercial or financial relationships that could be construed as a potential conflict of interest. The reviewer MM declared a shared affiliation, with no collaboration, with the authors CB and AS to the handling editor at the time of review.
